# [Corrigendum] Effects of nicorandil on p120 expression in the spinal cord and dorsal root ganglion of rats with chronic postsurgical pain

**DOI:** 10.3892/mmr.2025.13497

**Published:** 2025-03-19

**Authors:** Sai-Sai Huang, Su Cao, Cui E. Lu, Yi-Bin Qin, Jian-Ping Yang

Mol Med Rep 22: 4821–4827, 2020; DOI: 10.3892/mmr.2020.11546

Subsequently to the publication of the above paper, an interested reader drew to the authors’ attention that certain of the figures associated with four separate papers published by the same research group, and featuring some of the same authors, appeared to share immunofluorescence and graphical data. Upon analyzing the data independently in the Editorial Office, as far as the paper above was concerned, the diagram depicting the establishment of the skin/muscle incision and retraction (SMIR) model in rats, as featured in Fig. 1A on p. 4823, also appeared in one of the associated articles in the journal *Pain Research and Management* a couple of years afterwards.

Given that the sharing of the image in question has come to light, the authors wish to present an alternative version of Fig. 1, showing the original photograph depicting the SMIR model in Fig. 1A. This figure is shown below. All the authors agree with the publication of this corrigendum and are grateful to the Editor of *Molecular Medicine Reports* for allowing them the opportunity to publish this.

## Figures and Tables

**Figure 1.**
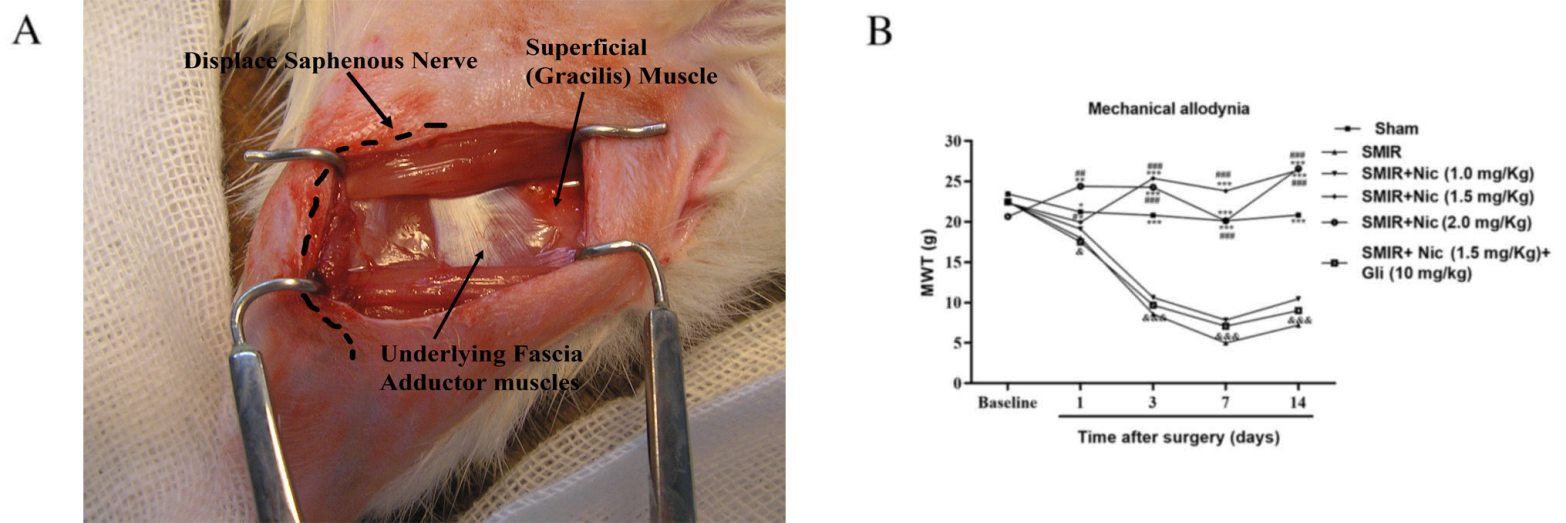
Establishment of the SMIR model in rats and detection of MWT. (A) SMIR model was established; the saphenous nerve was pulled and displaced. (B) Comparison of MWT in the sham, SMIR, Nic-treated groups and SMIR + Nic (1.5 mg/kg) + Gli group (n=6). *P<0.05, **P<0.01, ***P<0.001 vs. SMIR group; ^#^P<0.05, ^##^P<0.01, ^###^P<0.001 vs. SMIR + Nic (1.0 mg/kg) group; ^&^P<0.05, ^&&&^P<0.001 vs. SMIR + Nic (1.5 mg/kg) group. SMIR, skin/muscle incision and retraction; MWT, mechanical withdrawal threshold; Nic, nicorandil; Gli, glibenclamide.

